# Prognostic Value of N-Terminal Pro–B-Type Natriuretic Peptide for Risk Stratification in Adults With Fontan Palliation

**DOI:** 10.1016/j.cjcpc.2026.03.001

**Published:** 2026-03-13

**Authors:** Omar Abozied, William R. Miranda, Yogesh V.N. Reddy, Zeyad Kholeif, Heidi M. Connolly, Allan S. Jaffe, Alexander C. Egbe

**Affiliations:** Department of Cardiovascular Medicine, Mayo Clinic, Rochester, Minnesota, USA

**Keywords:** heart failure, mortality, prognostication

## Abstract

**Background:**

There are limited data about the prognostic role of N-terminal pro–B-type natriuretic peptide (NT-proBNP) in adults with Fontan palliation. The purpose of this study was to determine the correlates and the prognostic value of serial NT-proBNP assay for risk stratification in adults with Fontan palliation.

**Methods:**

This is a retrospective cohort study of adults with Fontan palliation who had ≥1 NT-proBNP measurement in the outpatient clinic (2003-2023). In patients with ≥3 NT-proBNP measurements, serial NT-proBNP was calculated as the mean of the first 3 measurements. The study outcome was death/transplant.

**Results:**

Of 369 patients (age 27 ± 8 years; 55% male) with NT-proBNP, 214 (58%) had serial measurements. The baseline level was 217 (82, 562) pg/mL, whereas the serial value was 261 (98, 715) pg/mL. The correlates of higher NT-proBNP levels were older age, atriopulmonary connection, atrial fibrillation, hepatorenal dysfunction, systemic ventricular dilation and systolic dysfunction, and higher Fontan pressures and pulmonary artery wedge pressure. Baseline and serial NT-proBNP levels were associated with death/transplant independent of anatomic indices, comorbidities, and hemodynamic indices. Serial NT-proBNP levels had superior prognostic value to single (baseline) NT-proBNP measurements, echocardiographic indices, cardiac magnetic resonance imaging indices, and cardiac catheterization indices.

**Conclusions:**

These findings have important clinical implications in the management of adults with Fontan palliation because NT-proBNP assay is inexpensive, readily available, and noninvasive, and hence ideal for longitudinal assessment. Further studies are required to determine whether NT-proBNP can be used as a surrogate marker to monitor response to therapeutic interventions in this population.

## Résumé

**Contexte:**

Les données sur le rôle pronostique du propeptide natriurétique de type B N-terminal (NT-proBNP) chez les adultes ayant subi une chirurgie palliative de Fontan sont limitées. L’objectif de cette étude consistait à déterminer les corrélats et la valeur pronostique du dosage en série du NT-proBNP pour la stratification du risque chez les adultes ayant subi une chirurgie palliative de Fontan.

**Méthodologie:**

Il s’agit d’une étude de cohorte rétrospective menée chez des adultes ayant subi une chirurgie palliative de Fontan qui s’étaient prêtés à au moins un dosage du NT-proBNP en clinique de jour (entre 2003 et 2023). Chez les patients pour lesquels on disposait d’au moins trois mesures du NT-proBNP, le résultat du dosage en série du NT-proBNP a été calculé en faisant la moyenne des trois premières mesures. Le critère d’évaluation de l’étude était le décès/la transplantation.

**Résultats:**

Parmi les 369 patients (âge : 27 ± 8 ans; 55 % d’hommes) dont le taux de NT-proBNP avait été mesuré, 214 (58 %) s’étaient prêtés à des dosages en série. Le taux initial était de 217 pg/ml (82 à 562), et le taux moyen, de 261 pg/ml (98 à 715). Les corrélats d’un taux de NT-proBNP accru étaient l’âge avancé, la connexion atrio-pulmonaire, la fibrillation auriculaire, la dysfonction hépato-rénale, la dilatation des ventricules et la dysfonction systolique, les pressions plus élevées après une chirurgie de Fontan et la pression capillaire pulmonaire bloquée. Les taux initial et moyen de NT-proBNP ont été associés au décès/à la transplantation, indépendamment des indices anatomiques, des affections concomitantes et des indices hémodynamiques. Les taux moyens de NT-proBNP ont eu une valeur pronostique supérieure aux mesures uniques (initiales) et aux indices fournis par l’échocardiographie, l’imagerie par résonance magnétique cardiaque et le cathétérisme cardiaque.

**Conclusions:**

Ces résultats ont des répercussions cliniques importantes pour la prise en charge des adultes ayant subi une chirurgie palliative de Fontan, car le dosage du NT-proBNP est un outil peu coûteux, accessible et non invasif, qui convient donc parfaitement à l’évaluation longitudinale. D’autres études s’imposent pour déterminer si le NT-proBNP peut être utilisé comme marqueur de substitution pour surveiller la réponse aux interventions thérapeutiques dans cette population.

Among 369 adults with Fontan palliation with N-terminal pro–B-type natriuretic peptide (NT-proBNP) measurements, we observed that higher NT-proBNP levels were associated with death/transplant, independent of anatomic indices, comorbidities, and hemodynamic indices. Serial NT-proBNP measurements provided superior prognostic performance to baseline (single) NT-proBNP measurements and conventional diagnostic testing such as echocardiographic, cardiac magnetic resonance imaging, and invasive hemodynamic indices. NT-proBNP assay is inexpensive, readily available, and noninvasive, and hence ideal for longitudinal assessment. Further studies are required to determine whether NT-proBNP can be used as a surrogate marker for monitoring response to therapy.

Adults with Fontan palliation have a high risk of cardiovascular and all-cause mortality compared with patients with congenital heart disease and biventricular circulation.^1^^,^^2^ The high risk of mortality in the Fontan population is related to the hemodynamic abnormalities inherent to the Fontan physiology.^3^, ^4^, ^5^ The absence of a subpulmonary ventricle results in an obligatory increase in central venous pressure, which in turn leads to systemic venous congestion and end-organ dysfunction affecting the liver, kidney, and gastrointestinal system.^3^, ^4^, ^5^, ^6^, ^7^ Furthermore, systemic ventricular systolic and diastolic function is common in this population, further exacerbating pulmonary venous congestion and impaired cardiac reserve.^8^

Cardiac catheterization (rest or exercise) is often used as the gold standard for hemodynamic assessment and risk stratification in adults with Fontan palliation because of the challenges associated with echo-Doppler hemodynamic assessment in this population.^8^, ^9^, ^10^, ^11^, ^12^ However, this method of hemodynamic assessment is invasive and not ideal for longitudinal monitoring. On the other hand, the assessment of cardiovascular biomarkers such as N-terminal pro–B-type natriuretic peptide (NT-proBNP) is noninvasive and can be used for prognostication in adults with congenital heart disease.^11^^,^^13^, ^14^, ^15^, ^16^, ^17^, ^18^ However, comparative analyses of the prognostic role of NT-proBNP relative to conventional invasive and noninvasive hemodynamic indices have not been systematically studied among patients with Fontan palliation. Furthermore, it is unknown whether serial measurements of NT-proBNP improved prognostication compared with a baseline measurement. The purpose of this study was to determine the correlates and the prognostic value of a single and serial NT-proBNP values for risk stratification in adults with Fontan palliation.

## Methods

### Study population

This is a retrospective cohort study of adults (aged ≥18 years) with Fontan palliation who received care at Mayo Clinic between January 1, 2003, and December 31, 2023. We reviewed the Mayo Adult Congenital Heart Disease (MACHD) Registry and identified consecutive patients who had ≥1 NT-proBNP measurement in the outpatient clinic.

### Data collection

The baseline encounter was the first outpatient encounter during which NT-proBNP was measured, and the NT-proBNP measured during the encounter was considered the baseline NT-proBNP. In patients with ≥3 NT-proBNP measurements, serial NT-proBNP was calculated as the mean of the first 3 measurements. In addition, we calculated absolute ΔNT-proBNP (NT-proBNP #3 – NT-proBNP #1) and relative ΔNT-proBNP ([NT-proBNP #3 – NT-proBNP #1]/NT-proBNP #1). Temporal increases in NT-proBNP were defined as a relative ΔNT-proBNP ≥1%.

The clinical assessments, echocardiograms, and laboratory data obtained during the baseline encounter were used to define the baseline clinical characteristics of the cohort. Echocardiographic data were retrieved from the MACHD Registry, and the procedural details for offline image analysis in MACHD Registry Imaging Core Laboratory have been described.^19^^,^^20^ In brief, 2-dimensional, Doppler, and speckle tracking echocardiography was performed according to contemporary guidelines, and offline image analysis was performed in all patients by research sonographers. Systemic ventricular volumes and ejection fraction were derived from the monoplane volumetric method from the apical 4-chamber window. Atrioventricular valve and aortic valve regurgitation was assessed by semiquantitative Doppler echocardiography. Similarly, cardiac magnetic resonance imaging (CMRI), cardiac catheterization, and treadmill cardiopulmonary exercise data obtained within 12 months from the baseline NT-proBNP were also retrieved from the MACHD Registry.

### Study objectives

The objectives of this study were as follows: (1) determine the correlates of baseline NT-proBNP and serial N-proBNP measurements; (2) assess the prognostic role of baseline and serial NT-proBNP measurements, the prognostic value of NT-proBNP was assessed as the relationship between NT-proBNP and the composite outcome of all-cause mortality and/or heart transplant (death/transplant); and (3) compare the prognostic performance of serial NT-proBNP measurements with baseline NT-proBNP as well as with conventional clinical indices such as echocardiographic indices (systemic ventricular ejection fraction and global longitudinal strain), cardiac catheterization indices (Fontan pressure and pulmonary artery wedge pressure), and exercise tests indices (%-predicted peak oxygen consumption).

### Statistical analysis

Data were presented as mean ± standard deviation, median (Q1, Q3), and count (%). The NT-proBNP data were log-transformed for all analyses because of skewed distribution. The correlates of baseline and serial NT-proBNP changes were assessed using linear regression analysis and mixed effect model analysis, respectively. First, univariable regression models were created to identify the correlates associated with NT-proBNP values. The variables used in the model include demographic indices (age, sex, and body mass index), anatomic indices (systemic ventricular morphology and heterotaxy), surgical history (type of Fontan connection, age of Fontan operation, cardiac implantable electronic device, and prosthetic valve implantation), comorbidities (atrial arrhythmias, chronic kidney disease, cirrhosis, and protein-losing enteropathy), laboratory indices (glomerular filtration rate, model for end-stage liver disease excluding international normalized ratio [MELD-XI]), cardiac medications, and echocardiographic indices (systemic ventricular volumes, ejection fraction, and valvular regurgitation severity). The covariates with *P* < 0.1 on univariable analysis were then used to create multivariable models using stepwise backward selection with *P* < 0.1 as the criterion for a variable to remain in the model. A subgroup analysis, incorporating invasive hemodynamic indices, was performed in the subset of patients who underwent cardiac catheterization using similar methods for covariate selection.

The cumulative incidence of death/transplant was estimated using Kaplan-Meier analysis in the subgroup of patients with serial NT-proBNP values (N = 214). In the absence of generally accepted cutoff points for NT-proBNP in Fontan physiology, we divided the patients with serial NT-proBNP into 3 subgroups using cutoff points (NT-proBNP <125 pg/mL and ≥300 pg/mL) associated with clinical outcomes from previous studies.^11^^,^^21^ Separate analyses were performed using the baseline and serial NT-proBNP measurements for categorization. The occurrence of death/transplant was assessed as a time-to-event outcome and ascertained by a review of medical records and the Accurint mortality database using the date of the third NT-proBNP assay as time 0. Furthermore, we dichotomized patients using the median relative ΔNT-proBNP, as well as using a temporal increase in relative ΔNT-proBNP (relative NT-proBNP ≥1%) vs others (relative NT-proBNP <1%).

The relationship between baseline NT-proBNP and death/transplant was assessed using Cox regression analysis, with covariate selection based on criteria similar to the regression models described above. The prognostic performance of NT-proBNP relative to conventional clinical indices was assessed using c-statistic comparison. All statistical analyses were performed with BlueSky Statistics software (version 7.10; BlueSky Statistics LLC, Chicago, IL) and JMP statistical software (version 17.1.0; JMP Statistical Discovery LLC, Cary, NC). A *P* value of <0.05 was considered to be statistically significant for all analyses.

## Results

### Baseline characteristics

A total of 369 patients met the inclusion criteria. The mean age at baseline encounter was 27 ± 8 years, and 204 (55%) were male. The underlying congenital heart disease diagnoses were tricuspid atresia (N = 114, 31%), double inlet left ventricle (N = 76, 21%), atrioventricular canal defect (N = 49, 13%), hypoplastic left heart syndrome (N = 43, 12%), double outlet right ventricle (N = 37, 10%), pulmonary atresia (n = 24, 7%), and others (N = 26, 7%). Of 369 patients, 236 (64%) had a systemic morphologic left ventricle. The types of Fontan connection were atriopulmonary (N = 120, 33%), lateral tunnel/intra-atrial conduit (N = 123, 33%), and extracardiac conduit connection (N = 126, 34%). Table 1 shows the baseline characteristics, comorbidities, and cardiovascular tests.

**Table 1:** Baseline characteristics

Characteristic	Value (N = 369)
Age (y)	27 ± 8
Male sex	204 (55)
Body surface area (m^2^)	1.80 ± 0.23
Body mass index (kg/m^2^)	24.7 ± 5.2
Systemic oxygen saturation (%)	92 (89, 94)
Anatomy	
Heterotaxy	37 (10)
Ventricular morphology	
LV	236 (64)
RV	110 (30)
Mixed	23 (6)
Surgical history	
Type of Fontan connection	
Atriopulmonary Fontan	120 (33)
Lateral tunnel/IAC Fontan	123 (33)
Extracardiac conduit Fontan	126 (34)
Age at Fontan operation (y)	4 (3, 7)
Prior history of Fontan conversion	96 (26)
CIED	116 (31)
AVV prosthesis	2 (0.5)
Aortic valve prosthesis	18 (5)
Comorbidities	
Atrial arrhythmias	151 (41)
Atrial flutter/tachycardia	108 (29)
Atrial fibrillation	62 (17)
CKD III-V	8 (2)
Cirrhosis	103 (28)
Protein losing enteropathy	36 (10)
Laboratory data	
NT-proBNP (pg/mL)	217 (82, 562)
Estimated GFR (mL/min/1.73 m^2^)	103 ± 24
Hemoglobin (g/dL)	14.9 ± 2.3
MELD-XI score	10.4 (9.4, 12.4)
Medications	
Diuretics	153 (42)
β-Blockers	149 (40)
ACEI/ARB	227 (62)
MRA	99 (27)
Echocardiographic indices	
Ventricular EDV index (mL/m^2^)	103 ± 39
Ventricular ESV index (mL/m^2^)	52 ± 36
Echo_EF (%)	51 ± 10
Ventricular EDA index (cm^2^/m^2^)	22 ± 6
Ventricular ESA index (cm^2^/m^2^)	15 ± 5
Echo_FAC (%)	35 ± 7
Stroke volume index (mL/m^2^)	45 ± 13
Ventricular GLS	16.8 ± 4.1
≥Moderate systemic AVV regurgitation	11 (3)
≥Moderate systemic aortic regurgitation	18 (5)
CMRI	N = 125
Ventricular EDV index (mL/m^2^)	90 ± 39
Ventricular ESV index (mL/m^2^)	46 ± 25
Stroke volume index (mL/m^2^)	44 ± 18
CMRI_EF (%)	49 ± 10
Exercise test	N = 220
Peak VO_2_ (mL/kg/min)	19.7 ± 6.7
Predicted peak VO_2_ (%)	49 ± 15
Cardiac catheterization data	N = 221
Fontan pressure (mm Hg)	14.2 ± 3.5
PAWP (mm Hg)	9.9 ± 3.4
PVR index (WU m^2^)	1.88 ± 1.02
Cardiac index (L/min/m^2^)	2.45 ± 0.67

Data are presented as mean ± standard deviation, median (Q1, Q3), or count (percent), as appropriate.

ACEI/ARB, angiotensin-converting enzyme inhibitor/angiotensin-II receptor blocker; AVV, atrioventricular valve; CIED, cardiac implantable electronic device; CKD, chronic kidney disease; CMRI, cardiac magnetic resonance imaging; EDA, end-diastolic area; EDV, end-diastolic volume; EF, ejection fraction; ESA, end-systolic area; ESV, end-systolic volume; FAC, fractional area change; GFR, glomerular filtration rate; GLS, global longitudinal strain; IAC, intra-atrial conduit; LV, left ventricle; MELD-XI, model for end-stage liver disease excluding international normalized ratio; MRA, mineralocorticoid receptor antagonist; NT-proBNP, N-terminal pro–B-type natriuretic peptide; PAWP, pulmonary artery wedge pressure; PVR, pulmonary vascular resistance; RV, right ventricle; VO_2_, oxygen consumption.

### Baseline NT-proBNP

The median baseline NT-proBNP was 217 (82, 562) pg/mL. Supplemental Table S1 shows the univariable model of the correlates of baseline NT-proBNP. On multivariable analysis, atriopulmonary connection, older age at Fontan operation, atrial fibrillation, chronic kidney disease, MELD-XI score, diuretic use, and larger echo-derived systemic ventricular volumes were associated with higher baseline NT-proBNP levels (Table 2).

**Table 2:** Multivariable linear regression models showing correlates of baseline NT-proBNP

	β ± SE	*P*
Patients with baseline NT-proBNP and echocardiographic data (N = 369)		
Surgical history		
AP (vs TCPC) Fontan	0.25 ± 0.01	<0.001
Age at Fontan operation (y)	0.03 ± 0.01	0.02
Comorbidities		
Atrial fibrillation	0.31 ± 0.08	<0.001
CKD III-V	0.14 ± 0.06	0.03
Laboratory data		
MELD-XI score	0.09 ± 0.02	<0.001
Medications		
Diuretics	0.23 ± 0.06	<0.001
Echocardiographic indices		
Ventricular EDV index (per 5 mL/m^2^)	0.07 ± 0.01	<0.001
Ventricular ESV index (per 5 mL/m^2^)	0.10 ± 0.01	<0.001
Patients with baseline NT-proBNP, echocardiographic, and catheterization data (N = 221)		
Surgical history		
AP (vs TCPC) Fontan	0.31 ± 0.09	<0.001
Comorbidities		
CKD III-V	0.21 ± 0.10	0.03
Laboratory data		
GFR (per 10 mL/min/1.73 m^2^)	–0.10 ± 0.03	0.002
Medications		
Diuretics	0.16 ± 0.08	0.04
Echocardiographic indices		
Ventricular EDV index (per 5 mL/m^2^)	0.07 ± 0.02	0.001
Ventricular ESV index (per 5 mL/m^2^)	0.13 ± 0.03	<0.001
Cardiac catheterization data		
Fontan pressure (mm Hg)	0.14 ± 0.04	0.001
PAWP (mm Hg)	0.08 ± 0.04	0.06

NT-proBNP was modeled as log NT-proBNP because of skewed distribution. See the Statistical analysis section for the criteria for constructing a regression model.

AP, atriopulmonary; β ± SE, β estimate ± standard error; CKD, chronic kidney disease; EDV, end-diastolic volume; ESV, end-systolic volume; GFR, glomerular filtration rate; MELD-XI, model for end-stage liver disease excluding international normalized; NT-proBNP, N-terminal pro–B-type natriuretic peptide; PAWP, pulmonary artery wedge pressure; TCPC, total cavopulmonary connection.

Of the 369 patients, 221 (60%) had cardiac catheterization data, and there was no significant difference in baseline NT-proBNP between patients with and without cardiac catheterization data (267 [119-622] vs 206 [83-453] pg/mL, *P* = 0.11), suggesting comparable subgroups. On multivariable analysis, atriopulmonary connection, chronic kidney disease, diuretic use, larger echo-derived systemic ventricular volumes, and higher Fontan pressure and pulmonary artery wedge pressure were associated with higher baseline NT-proBNP levels (Table 2).

### Serial NT-proBNP

Supplemental Figure S1 shows the number of patients with serial NT-proBNP measurements. The mean interval between NT-proBNP measurements was 13 ± 3 months. Supplemental Table S2 shows the univariable model of the correlates of serial NT-proBNP among the 214 patients with serial NT-proBNP measurements (ie, ≥3 NT-proBNP measurements). There was a temporal increase in NT-proBNP over time, leading to serial NT-proBNP of 261 (98, 715) pg/mL. Similar to the correlates for baseline NT-proBNP, we observed that older age, atriopulmonary connection, MELD-XI score, diuretic use, and larger echo-derived systemic ventricular volumes were associated with higher serial NT-proBNP (Table 3). Similar correlates of serial NT-proBNP, including higher Fontan pressure and pulmonary artery wedge pressure, were observed in the subgroup of patients with cardiac catheterization data (N = 150) (Table 3).

**Table 3:** Multivariable mixed model showing correlates of serial NT-proBNP

	β ± SE	*P*
Patients with serial NT-proBNP and echocardiographic data (N = 214)		
Age (per 5 y)	0.11 ± 0.03	0.003
Surgical history		
AP (vs TCPC) Fontan	0.28 ± 0.06	<0.001
Comorbidities		
Atrial fibrillation	0.22 ± 0.08	0.004
Laboratory data		
MELD-XI score	0.09 ± 0.02	<0.001
Medications		
Diuretics	0.24 ± 0.06	<0.001
Echocardiographic indices		
Ventricular EDV index (per 5 mL/m^2^)	0.06 ± 0.03	<0.003
Ventricular ESV index (per 5 mL/m^2^)	0.12 ± 0.02	<0.001
Patients with serial NT-proBNP, echocardiographic, and catheterization data (N = 150)		
Surgical history		
AP (vs TCPC) Fontan	0.34 ± 0.08	<0.001
Comorbidities		
Atrial fibrillation	0.23 ± 0.09	0.02
Laboratory data		
GFR (per 10 mL/min/1.73 m^2^)	–0.08 ± 0.03	0.006
Medications		
Diuretics	0.16 ± 0.07	0.02
Echocardiographic indices		
Ventricular EDV index (per 5 mL/m^2^)	0.05 ± 0.02	0.007
Ventricular ESV index (per 5 mL/m^2^)	0.10 ± 0.02	0.001
Cardiac catheterization data		
Fontan pressure (mm Hg)	0.12 ± 0.04	0.009
PAWP (mm Hg)	0.08 ± 0.04	0.01

NT-proBNP was modeled as log NT-proBNP because of skewed distribution. See the Statistical analysis section for the criteria for constructing a regression model.

AP, atriopulmonary; β ± SE, β estimate ± standard error; EDV, end-diastolic volume; ESV, end-systolic volume; GFR, glomerular filtration rate; MELD-XI, model for end-stage liver disease excluding international normalized ratio; NT-proBNP, N-terminal pro–B-type natriuretic peptide; PAWP, pulmonary artery wedge pressure; TCPC, total cavopulmonary connection.

Among the 214 patients with serial NT-proBNP measurements, absolute ΔNT-proBNP between NT-proBNP #1 and #3 was 24 (–64, 106) pg/mL, whereas the relative ΔNT-proBNP was 11% (–19, 82). There were 125 (58%) patients with a temporal increase in NT-proBNP (ie, relative ΔNT-proBNP ≥1%).

### Outcomes

The 369 patients were followed for 4.8 (1.6-8.4) years, and during this period, 71 patients (19%) died, and 22 (6%) patients underwent heart transplant, of whom 12 had combined heart-liver transplant. The composite outcome of death/transplant occurred in 89 (24%) patients. Using the baseline NT-proBNP for categorization, the 5-year cumulative incidence of death/transplant was 6%, 19%, and 33% for NT-proBNP <125 pg/mL, 125-299 pg/mL, and ≥300 pg/mL (*P* < 0.001), respectively (Fig. 1). Using the serial NT-proBNP for categorization, the 5-year cumulative incidence of death/transplant was 2%, 21%, and 34% for NT-proBNP <125 pg/mL, 125-299 pg/mL, and ≥300 pg/mL (*P* < 0.001), respectively (Fig. 1).

**Figure 1:**
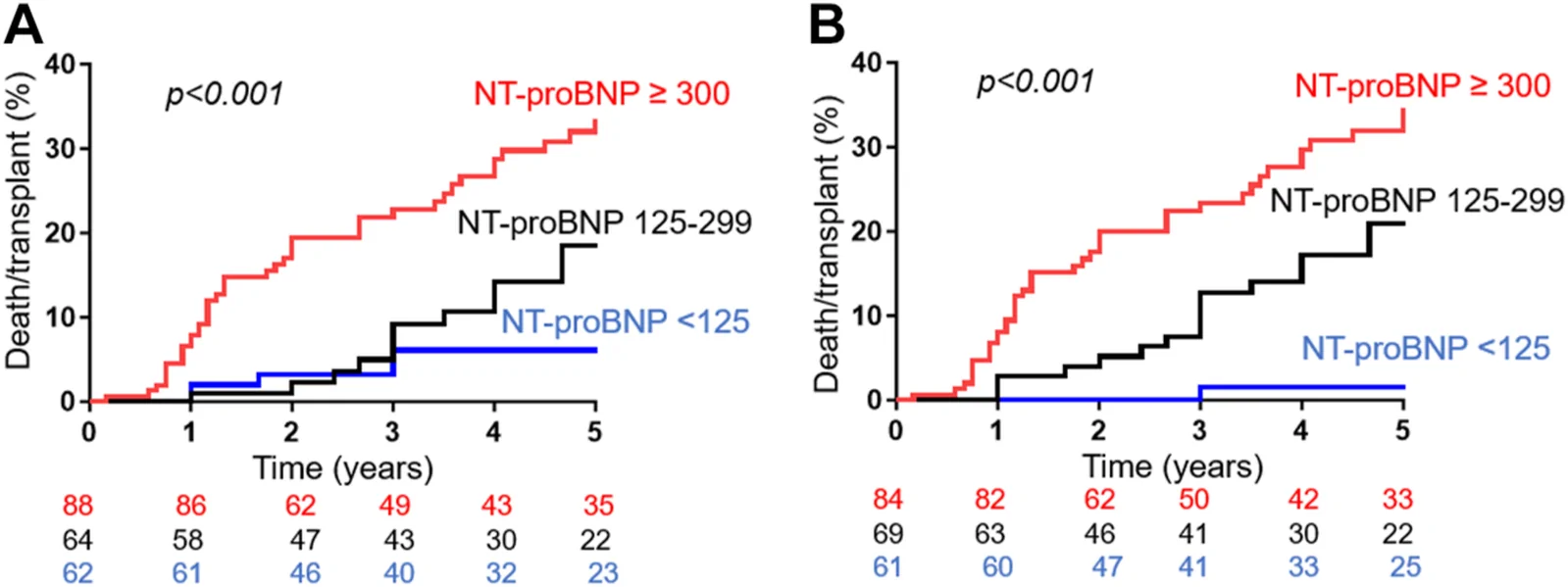
Kaplan-Meier curves comparing the cumulative incidence of death/transplant based on NT-proBNP levels for the patients with serial NT-proBNP levels defined as having ≥3 measurements (N = 214). Patients were stratified using the baseline (first) NT-proBNP measurement (**A**) and serial (mean of the measurements) (**B**). NT-proBNP, N-terminal pro–B-type natriuretic peptide.

Using relative ΔNT-proBNP for categorization, patients with relative ΔNT-proBNP above the median (ie, relative ΔNT-proBNP ≥11%) had a higher 5-year cumulative incidence of death/transplant compared with the patients with relative ΔNT-proBNP below the median (ie, relative ΔNT-proBNP <11%) (32% vs 12%, *P* < 0.001) (Fig. 2). Similarly, the patients with a temporal increase in NT-proBNP (ie, relative ΔNT-proBNP ≥1%) had a higher 5-year cumulative incidence of death/transplant compared with the patients without a temporal increase in NT-proBNP (ie, relative ΔNT-proBNP <1%) (29% vs 13%, *P* < 0.001) (Fig. 2).

**Figure 2:**
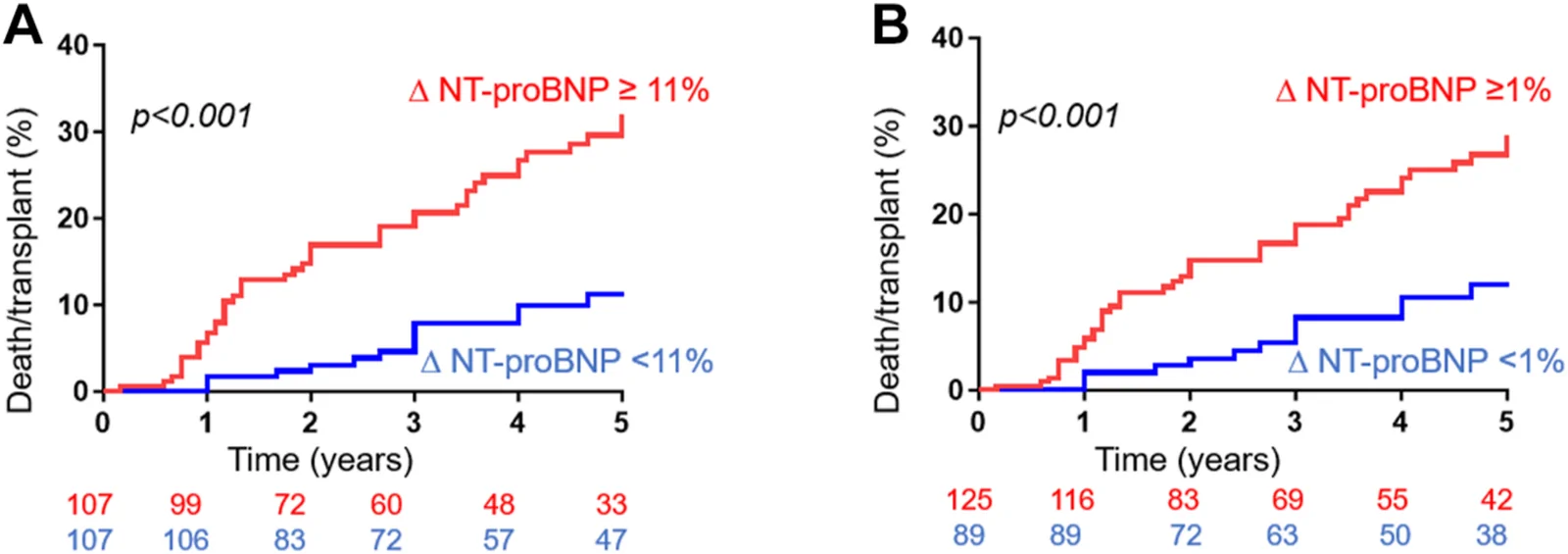
Kaplan-Meier curves comparing the cumulative incidence of death/transplant based on relative ΔNT-proBNP levels. Patients were stratified using the median relative ΔNT-proBNP levels (**A**) and using a temporal increase in ΔNT-proBNP levels (**B**). NT-proBNP, N-terminal pro–B-type natriuretic peptide.

Supplemental Table S3 shows the univariable Cox regression models of the predictors of death/transplant in the whole cohort and subgroup of patients with cardiac catheterization data. On multivariable analysis, the higher baseline NT-proBNP level was associated with higher risk of death/transplant in the entire cohort (hazard ratio: 1.39, 95% confidence interval [CI]: 1.09-1.77, *P* = 0.008) and in the subgroup of patients with cardiac catheterization data (hazard ratio: 1.32, 95% CI: 1.02-1.62, *P* = 0.01), after adjustment for demographic indices, surgical indices, comorbidities, laboratory indices, and hemodynamic indices (Table 4).

**Table 4:** Multivariable Cox regression models assessing the relationship between log NT-proBNP and death/transplant

	HR (95% CI)	*P*
Patients with baseline NT-proBNP and echocardiographic data (N = 369)		
Log NT-proBNP	1.39 (1.09-1.77)	0.008
Anatomy		
Heterotaxy	4.40 (1.87-10.4)	0.001
Non-LV (vs LV) morphology	0.46 (0.20-1.09)	0.08
Surgical history		
CIED	1.86 (1.16-2.95)	0.01
Comorbidities		
Atrial fibrillation	2.14 (1.13-4.04)	0.02
Laboratory data		
MELD-XI score	1.17 (1.09-1.25)	<0.001
Patients with baseline NT-proBNP, echocardiographic, and catheterization data (N = 221)		
Log NTproBNP	1.32 (1.02-1.62)	0.01
Demographic indices		
Age (per 5 y)	1.91 (1.3202.78)	<0.001
Systemic saturation (%)	0.96 (0.93-0.99)	0.001
Laboratory data		
MELD-XI score	1.66 (1.32-2.09)	<0.001
Cardiac catheterization data		
PAWP (mm Hg)	1.11 (1.01-1.23)	0.03

See the Statistical analysis section for the criteria for constructing a regression model.

CI, confidence interval; CIED, cardiac implantable electronic device; HR, hazard ratio; LV, left ventricle; MELD-XI, model for end-stage liver disease excluding international normalized ratio; NT-proBNP, N-terminal pro–B-type natriuretic peptide; PAWP, pulmonary artery wedge pressure.

### Prognostic performance of baseline NT-proBNP

Baseline NT-proBNP had superior prognostic performance to systemic ventricular ejection fraction (c-statistic difference: –0.118 [95% CI: –0.211 to –0.026], *P* = 0.012) and systemic ventricular global longitudinal strain (c-statistic difference: –0.116 [95% CI: –0.221 to –0.012], *P* = 0.029) but similar prognostic performance to Fontan pressures, pulmonary artery wedge pressure, and predicted peak oxygen (Table 5). In contrast, serial NT-proBNP had superior prognostic performance to systemic ventricular ejection fraction (c-statistic difference: –0.186 [95% CI: –0.278 to –0.094], *P* < 0.001), systemic ventricular global longitudinal strain (c-statistic difference: –0.142 [95% CI: –0.243 to –0.040], *P* = 0.006), Fontan pressures (c-statistic difference: –0.132 [95% CI: –0.239 to –0.024], *P* = 0.016), and pulmonary artery wedge pressure (c-statistic difference: –0.121 [95% CI: –0.226 to –0.017], *P* = 0.02) but similar prognostic performance to predicted peak oxygen consumption (Table 6).

**Table 5:** Prognostic performance of baseline NT-proBNP

	HR [95% CI]	*P*	C-statistic [95% CI]	C-statistic comparison [95% CI]	*P*
Log NT-proBNP (N = 369)	1.70 [1.47-1.96]	<0.0001	0.720 [0.654-0.786]	Reference	–
Echocardiographic indices (N = 369)					
Systemic ventricular EF (per 5%)	0.85 [0.77-0.93]	0.0005	0.601 [0.522-0.680]	–0.118 [–0.211 to –0.026]	0.012
Systemic ventricular GLS (per 1%)	0.94 [0.89-0.99]	0.015	0.589 [0.507-0.670]	–0.116 [–0.221 to –0.012]	0.029
Cardiac catheterization indices (N = 221)					
Fontan pressure (per 1 mm Hg)	1.14 [1.06-1.22]	0.0002	0.626 [0.539-0.712]	–0.055 [–0.151 to +0.041]	0.26
PAWP (per 1 mm Hg)	1.16 [1.08-1.25]	<0.0001	0.641 [0.556-0.727]	–0.040 [–0.146 to +0.067]	0.47
Exercise test indices (N = 221)					
Predicted peak VO_2_ (per 10%)	0.59 [0.48-0.72]	<0.0001	0.735 [0.665-0.804]	+0.012 [–0.062 to +0.102]	0.63
CMRI indices (N = 125)					
ESV index (per 5 mL/m^2^)	1.11 [0.97-1.27]	0.13	0.560 [0.343-0.778]	–0.162 [–0.423 to +0.099]	0.22
EF (per 5 mL/m^2^)	0.85 [0.64-1.12]	0.24	0.544 [0.320-0.768]	–0.178 [–0.451 to +0.094]	0.20

CI, confidence interval; CMRI, cardiac magnetic resonance imaging; EF, ejection fraction; ESV, end-systolic volume; GLS, global longitudinal strain; HR, hazard ratio; NT-proBNP, N-terminal pro–B-type natriuretic peptide; PAWP, pulmonary artery wedge pressure; VO_2_, oxygen consumption.

**Table 6:** Prognostic performance of serial NT-proBNP

	HR [95% CI]	*P*	C-statistic [95% CI]	C-statistic comparison [95% CI]	*P*
Log NT-proBNP (N = 214)	1.99 [1.65-2.40]	<0.0001	0.758 [0.687-0.829]	Reference	–
Echocardiographic indices (N = 214)					
Systemic ventricular EF (per 5%)	0.91 [0.80-1.04]	0.15	0.572 [0.483-0.661]	–0.186 [–0.278 to –0.094]	<0.0001
Systemic ventricular GLS (per 1%)	0.95 [0.89-1.00]	0.07	0.595 [0.511-0.678]	–0.142 [–0.243 to –0.040]	0.006
Cardiac catheterization indices (N = 150)					
Fontan pressure (per 1 mm Hg)	1.16 [1.07-1.26]	0.0004	0.631 [0.533-0.730]	–0.132 [–0.239 to –0.024]	0.016
PAWP (per 1 mm Hg)	1.19 [1.09-1.30]	<0.0001	0.642 [0.535-0.748]	–0.121 [–0.226 to –0.017]	0.02
Exercise test indices (N = 145)					
Predicted peak VO_2_ (per 10%)	0.48 [0.36-0.65]	<0.0001	0.762 [0.693-0.830]	+0.002 [–0.105 to +0.109]	0.97
CMRI indices (N = 91)					
ESV index (per 5 mL/m^2^)	1.08 [0.90-1.29]	0.41	0.498 [0.227-0.769]	–0.435 [–0.686 to –0.185]	0.007
EF (per 5 mL/m^2^)	0.93 [0.68-1.27]	0.63	0.481 [0.185-0.777]	–0.452 [–0.721 to –0.184]	0.001

CI, confidence interval; CMRI, cardiac magnetic resonance imaging; EF, ejection fraction; ESV, end-systolic volume; GLS, global longitudinal strain; HR, hazard ratio; NT-proBNP, N-terminal pro–B-type natriuretic peptide; PAWP, pulmonary artery wedge pressure; VO_2_, oxygen consumption.

## Discussion

In this study, we assessed the clinical correlates and prognostic value of baseline and serial assessments of NT-proBNP in adults with Fontan palliation. The main findings as follows: (1) Higher levels of NT-proBNP were associated with death/transplant, independent of anatomic indices, comorbidities, and hemodynamic indices. (2) Serial NT-proBNP measurements provided superior prognostic performance to baseline (single) NT-proBNP measurements and conventional clinical indices such as echocardiographic indices, invasive hemodynamic indices, and CMRI indices. (3) The correlates of higher NT-proBNP levels were older age, atriopulmonary connection, atrial fibrillation, hepatorenal dysfunction, systemic ventricular dilation and systolic dysfunction, and higher Fontan pressures and pulmonary artery wedge pressures.

NT-proBNP is a widely used biomarker in the diagnosis and management of heart failure.^21^^,^^22^ NT-proBNP is the inactive fragment cleaved from proBNP in response to myocardial wall stress, making it a valuable indicator of ventricular dysfunction and congestion.^21^^,^^22^ Similar findings have been reported in Fontan physiology whereby higher NT-proBNP levels were associated with systemic ventricular dilation, systemic ventricular dysfunction, and atriopulmonary connection.^14^^,^^15^^,^^18^ Furthermore, NT-proBNP >300 pg/mL is associated with higher Fontan pressures and pulmonary artery wedge pressures during exercise, both of which are known markers of Fontan failure and mortality.^11^^,^^13^ The results of the current study are consistent with these previous studies supporting NT-proBNP as a marker of venous congestion and systemic ventricular dysfunction.^11^^,^^13^ In addition, our data augment the literature by providing criteria based on changes in NT-proBNP, which may be helpful clinically.

The American and European guidelines for the management of heart failure recommend the use of NT-proBNP for risk stratification in patients with acute as well as chronic heart failure.^21^^,^^22^ This is based on robust data linking rising NT-proBNP levels with worsening heart failure and cardiovascular mortality in patients with acquired heart disease.^21^, ^22^, ^23^, ^24^ However, data supporting the role of NT-proBNP for prognostication in adults with Fontan palliation are sparse. Lin et al.^17^ observed that a high NT-proBNP level was associated with an adverse outcome among 91 pediatric patients undergoing Fontan operation, whereas Van Hassel et al.^16^ demonstrated that a higher NT-proBNP level was associated with deterioration in renal function. These prior studies were limited by small sample size and were predominantly based on pediatric patients, which limits generalizability to the adult Fontan population.^16^^,^^17^ The current study demonstrates a robust association between NT-proBNP and mortality based on a large sample of a well-characterized cohort of adults with Fontan palliation. Furthermore, the study demonstrated that serial NT-proBNP measurement had superior prognostic performance to a single baseline measurement and was also superior to invasive Fontan hemodynamics, which is considered the gold standard of hemodynamic assessment in Fontan physiology. The prognostic performance of serial NT-proBNP was comparable only to predicted peak oxygen consumption, which is a validated prognostic marker for outcomes in this population.^25^^,^^26^ We postulate that the superior prognostic performance of serial measurements to single baseline measurement suggests that an average of multiple measurements is more representative of the patient’s physiology than a single baseline measurement. Furthermore, serial NT-proBNP incorporates longitudinal changes in NT-proBNP levels over time, which in turn reflects worsening disease condition or improvement in response to therapy, as evidenced by the observed differences in outcomes based on relative ΔNT-proBNP (Fig. 2). These findings support serial measurements of NT-proBNP in clinical practice because of superior prognostic performance (relative to other conventional clinical risk stratification tools) and because NT-proBNP assay is inexpensive, readily available, and noninvasive. Further studies are required to determine whether NT-proBNP can be used as a surrogate marker to assess response to therapeutic interventions in this population.

### Limitations

This is a retrospective single-center study conducted in a tertiary adult congenital heart disease center, and it is therefore prone to selection and ascertainment bias. We relied on echocardiography for the assessment of systemic ventricular volume and ejection fraction because CMRI data were not available in all patients. Finally, although we adjusted for differences in baseline characteristics, we did not account for the confounding effect of any therapeutic interventions during follow-up.

## Conclusions

Higher NT-proBNP levels were associated with death/transplant in adults with Fontan palliation. Serial NT-proBNP measurement had a superior prognostic value to single (baseline) NT-proBNP measurements, echocardiographic indices, and invasive hemodynamic indices. These findings support serial measurements of NT-proBNP for risk stratification considering the superior prognostic performance and relative ease of implementation in routine clinical practice. Further studies are required to determine whether medical and surgical interventions would result in improvement in NT-proBNP levels and clinical outcomes, and whether NT-proBNP can be used as a surrogate marker to assess response to therapeutic interventions in this population.

## Ethics Statement

The research reported in this paper adhered to STrengthening the Reporting of OBservational studies in Epidemiology (STROBE) guidelines.

## Patient Consent

The authors confirm that patient consent is not applicable to this article. The study was based on retrospective chart review.

## Funding Sources

ACE is supported by 10.13039/100000050National Heart, Lung, and Blood Institute (grants: R01 HL158517, R01 HL160761, and R01 HL162830). The Mayo Adult Congenital Heart Disease Registry is supported by the Al-Bahar Research grant.

## Disclosures

ASJ has been consulting for most of the major *in vitro* diagnostic companies who make N-terminal pro–B-type natriuretic peptide assays. The other authors have no conflicts of interest to disclose.

## Footnotes

To access the supplementary material accompanying this article, visit *CJC Pediatric and Congenital Heart Disease* at https://www.cjcpc.ca/ and at https://doi.org/10.1016/j.cjcpc.2026.03.001.
