# Supplementary material for: Prognostic Value of N-Terminal Pro–B-Type Natriuretic Peptide for Risk Stratification in Adults With Fontan Palliation
Source: CJC Pediatr Congenit Heart Dis. 2026 Mar 13;5(5):282–90. doi: 10.1016/j.cjcpc.2026.03.001 (PMC13712465; doi:10.1016/j.cjcpc.2026.03.001)
Supplement: Supplementary file 1 [file mmc1.docx]

**Supplemental Figure S1: Title: Bar graphs showing the total number of patients (as number of patients with cardiac catheterization data [red] and no cardiac catheterization data [blue]) stratified by number of NT-proBNP measurements**

**Supplemental Table S1: Univariable Linear Regression Model Showing Correlates of Baseline NT-proBNP.**

|  | **Univariable analysis** | |
| --- | --- | --- |
|  | **β±SE** | **P** |
| Age, per 5 years | 0.19±0.04 | <0.001 |
| Male sex | 0.02±0.07 | 0.74 |
| Body mass index, kg/m^2^ | 0.01±0.01 | 0.59 |
|  |  |  |
| **Anatomy** |  |  |
| Heterotaxy | 0.29±0.11 | 0.01 |
| Non-LV (vs LV) morphology | 0.10±0.07 | 0.16 |
|  |  |  |
| **Surgical history** |  |  |
| AP (vs TCPC) Fontan | 0.34±0.07 | <0.001 |
| Age at Fontan operation, years | 0.07±0.01 | <0.001 |
| Prior history of Fontan conversion | 0.04±0.08 | 0.66 |
| CIED | 0.15±0.07 | 0.05 |
| AV valve prosthesis | 0.23±0.47 | 0.62 |
| Aortic valve prosthesis | 0.30±0.16 | 0.06 |
|  |  |  |
| **Comorbidities** |  |  |
| Atrial flutter/tachycardia | 0.28±0.08 | <0.001 |
| Atrial fibrillation | 0.49±0.09 | <0.001 |
| CKD III-V | 0.82±0.24 | <0.001 |
| Cirrhosis | 0.17±0.08 | 0.03 |
| Protein losing enteropathy | 0.16±0.12 | 0.18 |
|  |  |  |
| **Laboratory data** |  |  |
| GFR, per 10 ml/min/1.73 m^2^ | -0.16±0.03 | <0.001 |
| MELD-XI score | 0.15±0.02 | <0.001 |
|  |  |  |
| **Medications** |  |  |
| Diuretics | 0.46±0.07 | <0.001 |
| ACEI/ARB | 0.01±0.07 | 0.86 |
| MRA | 0.19±0.08 | 0.02 |
|  |  |  |
| **Echocardiographic indices** |  |  |
| Ventricular EDV index, per 5 ml/m^2^ | 0.02±0.01 | 0.003 |
| Ventricular ESV index, per 5 ml/m^2^ | 0.05±0.01 | <0.001 |
| Echo_EF, per 5% | -0.23±0.32 | <0.001 |
| ≥Moderate AVV regurgitation | 0.17±0.21 | 0.21 |
| ≥Moderate aortic regurgitation | 0.30±0.16 | 0.06 |
|  |  |  |
| **Cardiac catheterization data** |  |  |
| Fontan pressure, mmHg | 0.12±0.02 | <0.001 |
| PAWP, mmHg | 0.10±0.03 | <0.001 |
| PVR index, WU*m2 | 0.16±0.09 | 0.07 |
| Cardiac index, l/min/m^2^ | -0.19±0.13 | 0.12 |

**Abbreviations**: ACEI/ARB: Angiotensin-converting enzyme inhibitor/angiotensin-II receptor blocker; AVV: Atrioventricular valve; CKD: Chronic kidney disease; CIED: Cardiac implantable electronic device; EF: Ejection fraction; EDV: End-diastolic volume; ESV: End systolic volume; GFR: Glomerular filtration rate; LV: Left ventricle; MRA: Mineralocorticoid receptor antagonist; MELD-XI: Model for end-stage liver disease excluding international normalized ratio; NTproBNP: N terminal pro hormone brain natriuretic peptide; PAWP: Pulmonary artery wedge pressure; PVR: Pulmonary vascular resistance; TCPC: Total cavopulmonary connection. β±SE: beta estimate ± standard error

**Footnote**: NT-proBNP was modeled as log NT-proBNP because of skewed distribution. See statistical analysis section for the criteria for constructing regression model.

**Supplemental Table S2: Univariable Mixed Models Showing Correlates of Serial NT-proBNP**

|  | **Univariable analysis** | |
| --- | --- | --- |
|  | **β±SE** | **P** |
| Age, per 5 years | 0.16±0.05 | 0.003 |
| Male sex | 0.02±0.09 | 0.81 |
| Body mass index, kg/m^2^ | -0.02±0.02 | 0.23 |
|  |  |  |
| **Anatomy** |  |  |
| Heterotaxy | 0.08±0.15 | 0.61 |
| Non-LV (vs LV) morphology | -0.01±0.09 | 0.88 |
|  |  |  |
| **Surgical history** |  |  |
| AP (vs TCPC) Fontan | 0.49±0.09 | <0.001 |
| Age at Fontan operation, years | 0.05±0.02 | <0.001 |
| Prior history of Fontan conversion | 0.07±0.11 | 0.46 |
| CIED | 0.16±0.10 | 0.11 |
| AV valve prosthesis | -0.26±0.47 | 0.57 |
| Aortic valve prosthesis | 0.34±0.20 | 0.09 |
|  |  |  |
| **Comorbidities** |  |  |
| Atrial flutter/tachycardia | 0.18±0.09 | 0.06 |
| Atrial fibrillation | 0.41±0.11 | <0.001 |
| CKD III-V | 0.60±0.29 | 0.05 |
| Cirrhosis | 0.06±0.10 | 0.55 |
| Protein losing enteropathy | 0.16±0.14 | 0.28 |
|  |  |  |
| **Laboratory data** |  |  |
| GFR, per 10 ml/min/1.73 m^2^ | -0.15±0.04 | <0.001 |
| MELD-XI score | 0.10±0.03 | <0.001 |
|  |  |  |
| **Medications** |  |  |
| Diuretics | 0.37±0.09 | <0.001 |
| ACEI/ARB | 0.06±0.10 | 0.55 |
| MRA | 0.11±0.10 | 0.25 |
|  |  |  |
| **Echocardiographic indices** |  |  |
| Ventricular EDV index, per 5 ml/m^2^ | 0.02±0.01 | 0.02 |
| Ventricular ESV index, per 5 ml/m^2^ | 0.04±0.01 | 0.001 |
| Echo_EF, per 5% | -0.22±0.04 | <0.001 |
| ≥Moderate systemic AVVR | 0.18±0.27 | 0.52 |
| ≥Moderate aortic regurgitation | 0.36±0.20 | 0.08 |
|  |  |  |
| **Cardiac catheterization data** |  |  |
| Fontan pressure, mmHg | 0.12±0.02 | <0.001 |
| PAWP, mmHg | 0.10±0.03 | <0.001 |
| PVR index, WU*m2 | 0.16±0.09 | 0.07 |
| Cardiac index, l/min/m^2^ | -0.19±0.13 | 0.12 |

**Abbreviations**: ACEI/ARB: Angiotensin-converting enzyme inhibitor/angiotensin-II receptor blocker; AVV: Atrioventricular valve; CKD: Chronic kidney disease; CIED: Cardiac implantable electronic device; EF: Ejection fraction; EDV: End-diastolic volume; ESV: End systolic volume; GFR: Glomerular filtration rate; LV: Left ventricle; MRA: Mineralocorticoid receptor antagonist; MELD-XI: Model for end-stage liver disease excluding international normalized ratio; NTproBNP: N terminal pro hormone brain natriuretic peptide; PAWP: Pulmonary artery wedge pressure; PVR: Pulmonary vascular resistance; TCPC: Total cavopulmonary connection. β±SE: beta estimate ± standard error

**Footnote**: NT-proBNP was modeled as log NT-proBNP because of skewed distribution. See statistical analysis section for the criteria for constructing regression model.

**Supplemental Table S3: Univariable Cox Regression Models Showing Correlates of Death/transplant.**

|  | **Univariable analysis** | |
| --- | --- | --- |
|  | **HR (95% CI)** | **P** |
| Log NT-proBNP | 1.82 (1.51-1.51) | <0.001 |
|  |  |  |
| **Demographic indices** |  |  |
| Age, per 5 years | 1.35 (1.18-1.54) | <0.001 |
| Male sex | 0.84 (0.47-1.48) | 0.54 |
| Systemic saturation, % | 0.98 (0.96-1.01) | 0.08 |
|  |  |  |
| **Anatomy** |  |  |
| Heterotaxy | 2.95 (1.46-5.96) | 0.006 |
| Non-LV (vs LV) morphology | 0.44 (0.19-1.07) | 0.07 |
|  |  |  |
| **Surgical history** |  |  |
| AP (vs TCPC) Fontan | 2.00 (1.12-3.53) | 0.02 |
| Age at Fontan operation, years | 1.07 (1.03-1.09) | <0.001 |
| Prior history of Fontan conversion | 1.13 (0.61-1.92) | 0.76 |
| CIED | 1.25 (1.27-3.99) | 0.001 |
| AV valve prosthesis | 1.47 (0.54-4.73) | 0.61 |
| Aortic valve prosthesis | 1.51 (0.59-4.02) | 0.49 |
|  |  |  |
| **Comorbidities** |  |  |
| Atrial flutter/tachycardia | 1.53 (0.83-2.79) | 0.18 |
| Atrial fibrillation | 3.46 (1.93-6.20) | <0.001 |
| CKD III-V | 3.45 (1.06-11.1) | 0.03 |
| Cirrhosis | 2.08 (1.14-3.78) | 0.02 |
| Protein losing enteropathy | 1.56 (0.91-3.05) | 0.36 |
|  |  |  |
| **Laboratory data** |  |  |
| GFR, per 10 ml/min/1.73 m^2^ | 0.77 (0.70-0.85) | <0.001 |
| MELD-XI score | 1.26 (1.19-1.33) | <0.001 |
|  |  |  |
| **Medications** |  |  |
| Diuretics | 2.73 (1.49-4.99) | 0.01 |
| ACEI/ARB | 0.52 (0.29-0.93) | 0.02 |
| MRA | 0.88 (0.49-1.39) | 0.42 |
|  |  |  |
| **Echocardiographic indices** |  |  |
| Ventricular EDV index, per 5 ml/m^2^ | 1.03 (0.98-1.08) | 0.33 |
| Ventricular ESV index, per 5 ml/m^2^ | 1.03 (1.01-1.05) | 0.01 |
| Echo_EF, per 5% | 0.83 (0.75-0.94) | 0.002 |
| ≥Moderate AVV regurgitation | 1.21 (0.46-4.80) | 0.81 |
| ≥Moderate aortic regurgitation | 0.63 (0.41-6.31) | 0.49 |
|  |  |  |
| **Cardiac catheterization data** |  |  |
| Fontan pressure, mmHg | 1.14 (1.06-1.22) | <0.001 |
| PAWP, mmHg | 1.16 (1.08-1.25) | <0.001 |
| PVR index, WU*m2 | 0.99 (0.73-1.27) | 0.92 |
| Cardiac index, l/min/m^2^ | 1.18 (0.76-1.77) | 0.45 |

**Abbreviations**: ACEI/ARB: Angiotensin-converting enzyme inhibitor/angiotensin-II receptor blocker; AVV: Atrioventricular valve; CI: Confidence interval; CKD: Chronic kidney disease; CIED: Cardiac implantable electronic device; EF: Ejection fraction; EDV: End-diastolic volume; ESV: End systolic volume; GFR: Glomerular filtration rate; HR: Hazard ratio; LV: Left ventricle; MRA: Mineralocorticoid receptor antagonist; MELD-XI: Model for end-stage liver disease excluding international normalized ratio; NTproBNP: N terminal pro hormone brain natriuretic peptide; PAWP: Pulmonary artery wedge pressure; PVR: Pulmonary vascular resistance; TCPC: Total cavopulmonary connection.

**Footnote**: See statistical analysis section for the criteria for constructing regression model.
